# The progression of the vertebral body bruise associated with a spinal fracture

**DOI:** 10.1186/s12891-022-05405-7

**Published:** 2022-05-13

**Authors:** Young-Woo Kim, Seong-Hwan Moon, Sung Hye Koh, Ki Tae Kim, Won Yong Yoon, Jeong Hwan Lee, Seonghyeon Kim, Paul S. Sung, Moon Soo Park

**Affiliations:** 1 Department of Orthopaedic Surgery, Hallym University Dongtan Sacred Heart Hospital, Medical College of Hallym University, 7, Keunjaebong-gil, Hwaseong-si, Gyeonggi-do 18450 Republic of Korea; 2 Department of Orthopaedic Surgery, Yonsei University College of Medicine, 50-1 Yonsei-ro, Seodaemun-gu, Seoul, 03722 Republic of Korea; 3 Department of Radiology, Hallym University Sacred Heart Hospital, Medical College of Hallym University, 22 Gwanpyeong-ro 170 beon-gi, Dongan-gu, Anyang-si, Gyeonggi-do 14068 Republic of Korea; 4 Department of Orthopaedic Surgery, Hallym University Sacred Heart Hospital, Medical College of Hallym University, 22 Gwanpyeong-ro 170 beon-gi, Dongan-gu, Anyang-si, Gyeonggi-do 14068 Republic of Korea; 5 Department of Physical Therapy, Indiana Wesleyan University, 4201 South Washington Street, Marion, IN 46953 USA

**Keywords:** spine, Trauma, MRI, Vertebral body bruise, Progression

## Abstract

**Background:**

Advances in magnetic resonance imaging (MRI) have made it possible to find the vertebral body bruise (VBB), which was not found in computed tomography (CT) after trauma. There has been only one study with adult patients about whether traumatic VBB will cause a collapse of the vertebral body or not. The purpose is to elucidate the progression of VBB in non-osteoporotic adult patients and to identify the possible factors influencing the progression.

**Method:**

The VBB was defined on MRI as band-like or diffuse zones of high signal intensity on T2-weighted sequences without fracture of the cortex based on CT. The study population with traumatic VBB associated with non-osteoporotic spinal fracture was composed of 15 females and 21 males. The minimal follow-up period was 6 months. The ratio of anterior to posterior heights of the VBB, the ratio of anterior heights of the VBB to the average of those of cranial and caudal adjacent vertebral bodies, the anterior wedge angle of the VBB, and the focal angle around the VBB were compared between the initial and final visits. We evaluated the age of the patients, the C2 plumb line distance, the regional location of VBB, the etiology of VBB, and the treatment methods of the fractures as possible risk factors influencing the progression.

**Results:**

There was no difference in the ratios and angles between the initial and final visits. The differences in the ratios and angles between the initial and final visits were not dependent on the possible risk factors. The anterior superior area is the most common in the distribution of VBB.

**Conclusions:**

Unlike compression fractures, the vertebral body with traumatic VBB found in adult patients with non-osteoporotic spinal fractures of AO classification A or B types did not develop collapse. In clinical practice, it is reasonable to diagnose it as a spinal fracture rather than a VBB if the collapse of a possible VBB occurs.

## Introduction

Patients with back pain after trauma undergo various tests such as radiographs, computed tomography (CT), and magnetic resonance imaging (MRI). MRI is a useful test that can detect not only the damage of ligaments and tendons, but also bony problems such as fractures.

MRI can identify traumatic vertebral body bruises (VBB), which cannot be confirmed by radiographs or CT [1, 2]. The VBB is defined on an MRI as band-like or diffuse zones of high signal intensity on T2-weighted sequences without associated fracture of the cortex [3]. The traumatic VBB is common in 57% of patients who underwent whole spine MRI due to spinal fractures [4]. As the use of MRI became more common, the traumatic VBB encounters became more common in clinical practice.

The traumatic VBB is usually treated with conservative methods [3, 5]. The conservative treatment is based on the progression of traumatic VBB. However, there has been only one study about the progression of traumatic VBB in adult patients [5].

The purpose is to elucidate the progression of VBB in non-osteoporotic adult patients and to identify the possible risk factors influencing the progression.

## Material and methods

The current study was retrospective with consecutive patients between January 2014 and December 2016 at a single tertiary hospital. The patients were included if they had undergone radiographs, CT, MRI of the spine, bone densitometry, and had the final diagnosis of non-osteoporotic spinal fracture. Patients were excluded if they had a diagnosis of infection, tumor, or a history of previous spinal surgeries. Patients younger than 20 years old were also excluded.

**Fig. 1:**
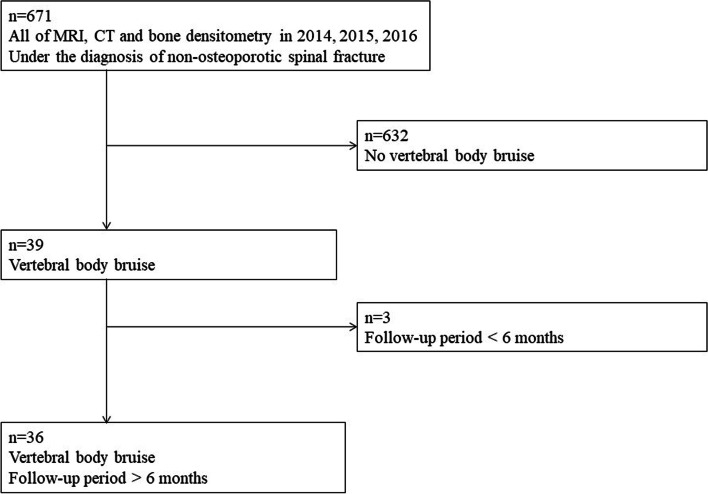
Study population

The 671 patients in this study had undergone the radiographs, CT, MRI of the spine, and bone densitometry to have the diagnosis of non-osteoporotic spinal fracture (Fig. 1). Thirty-nine patients (5.8%) were identified with having VBB on the other spinal level of the vertebral body with no cortical fractures based on the spinal CT. The final study population with VBB included thirty-six patients with the exclusion of three patients due to the follow-up period of shorter than 6 months (Fig. 1).

**Fig. 2:**
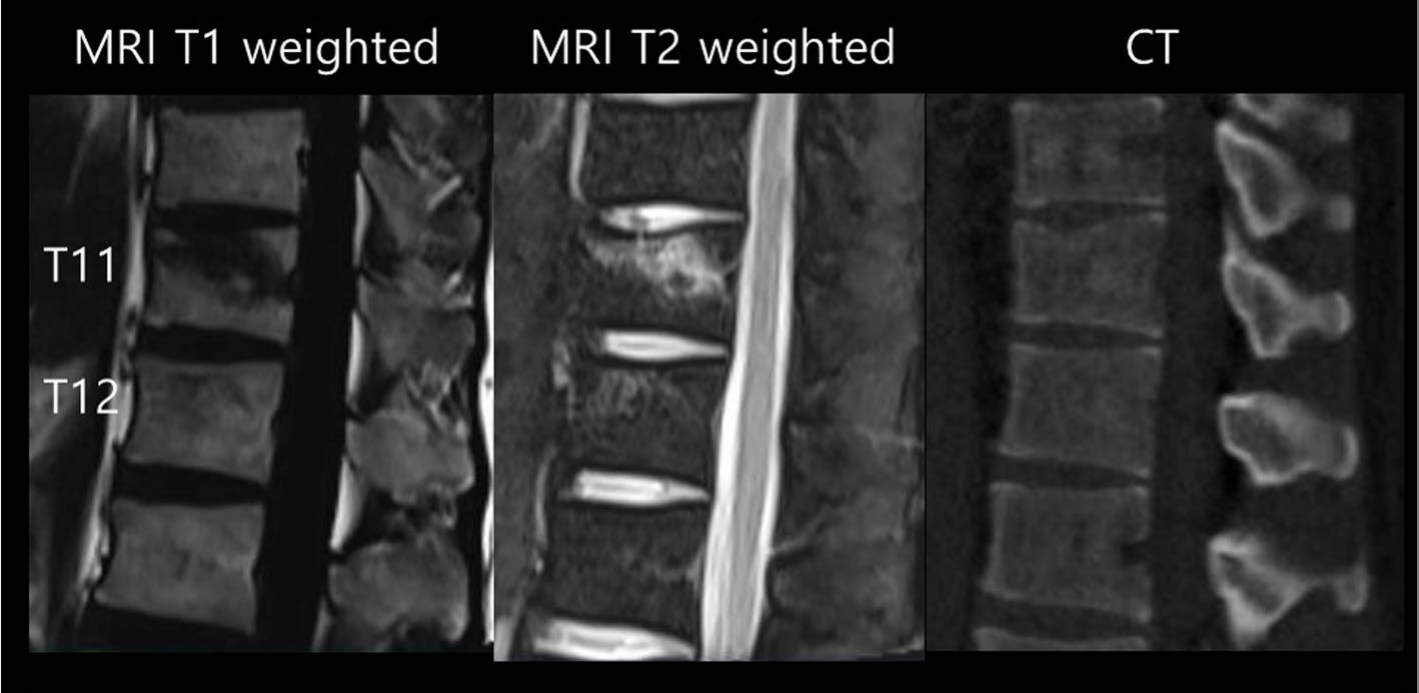
VBB found at T12 of the thirty-three-years-old patient with T11 compression fracture of AO classification A1 type

All radiographs, CT, MRI, and bone densitometry were taken within two weeks after trauma. All MRIs were obtained using a 1.5-T superconductive imager (Intera, Koninklijke Philips Electronics N.V.) under the following settings: sagittal T1-weighted spin-echo, sagittal fat-saturated T2-weighted fast spin-echo imaging, and axial T1 weighted spin-echo sequences. They were retrospectively evaluated to find the traumatic VBB in the other spinal levels except for the fractured spinal levels. VBB is defined to be hypointense on T1-weighted and to be hyperintense on T2-weighted sequences on the MRI (Fig. 2). VBB has an intact cortex based on CT (Fig. 2). An experienced spine surgeon and an experienced radiologist assessed the radiographs, the CT, and MRI from the time of injury to identify the VBB. When they had different opinions, they met to reach a similar conclusion after careful discussion.

The final study population with VBB included 15 females and 21 males, aged 33–76 years old (mean 50.5 ± 16.6 years old). The etiologies of VBB were slipping (twenty-six patients, 72.2%), falling (four patients, 11.1%), and traffic accidents (six patients, 16.7%). VBB was found in the most vertebral bodies from C2 to L5 on the MRI (Table 1). Ten VBB (27.8%) were found in the cervical, twenty-two (61.1%) in the thoracic, and four (11.1%) in the lumbar spine (Table 1). Fifteen out of thirty-six patients with VBB were related with fractures of multiple spinal levels (41.7%), and twenty-one out of thirty-six patients with VBB were related with fractures of single spinal level (58.3%, Table 1). The fractures were composed of AO classification A1 type in twenty-five patients (69.4%), A3 type in three patients (8.3%), A4 type in one patient (2.8%), B1 type in one patient (2.8%), B2 type in five patients (13.9%), and B3 type in one patient (2.8%). Six patients with VBB had undergone operations (16.7%), and VBB was not present in the instrumented levels of them. The other thirty patients with VBB (83.3%) received conservative treatment with a Philadelphia brace or a thoracolumbar orthosis for three months. The mean body mass index (BMI) of the final study population was 23.0 ± 3.2 kg/m^2^ (range: 16.9–31.0 kg/ m^2^). The mean T score of bone mineral density was -1.30 ± 1.08. The mean follow-up period was 14.6 ± 18.0 months (6–72 months).

**Table 1:** Spinal level with traumatic vertebral body bruises (VBB) and fractures. The VBBs combined with the fractures of multiple spinal levels are underlined

Spinal level of VBB	Number	Percentage	Spinal level of fractures
C2	1	2.8%	C7
C4	1	2.8%	C5
C5	2	5.6%	C7 / T2, T3, T4
C6	3	8.3%	C5 / C7 / T2, T3, T4
C7	3	8.3%	T1 / T2, T3 / T2, T3, T4
T1	2	5.6%	T12 / T4, T7, T10, T11
T2	2	5.6%	T3, T4 / C4, T4, T7, T10, T11
T3	2	5.6%	T2 / T12, L1, L4
T4	1	2.8%	L2
T5	1	2.8%	T10, T12
T6	1	2.8%	T12, L4
T7	4	11.1%	C4 / T11 / T2 / T4, T10, T11
T8	2	5.6%	T11 / T12
T9	2	5.6%	T12 / T10, T12
T10	3	8.3%	T11 / T12 / T12
T12	2	5.6%	T11 / T10, L5
L3	2	5.6%	L2 / T10, T12
L4	1	2.8%	L1
L5	1	2.8%	T12, L2

**Fig. 3:**
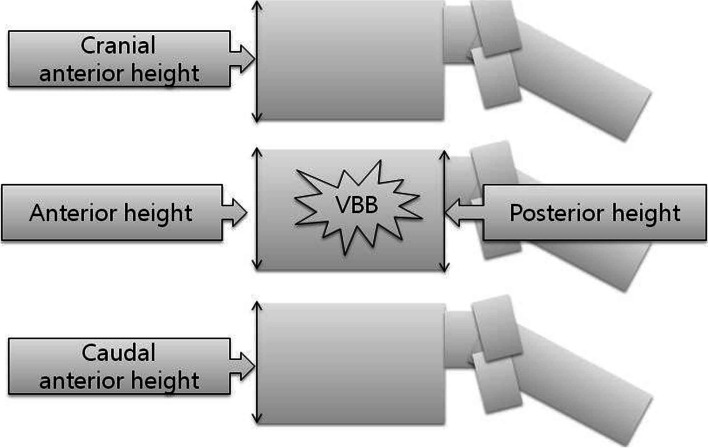
The ratio of anterior to posterior heights of VBB (AP ratio) and the ratio of anterior heights of the VBB to the average of those of cranial and caudal adjacent vertebral bodies (ratio to adjacent spines)

**Fig. 4:**
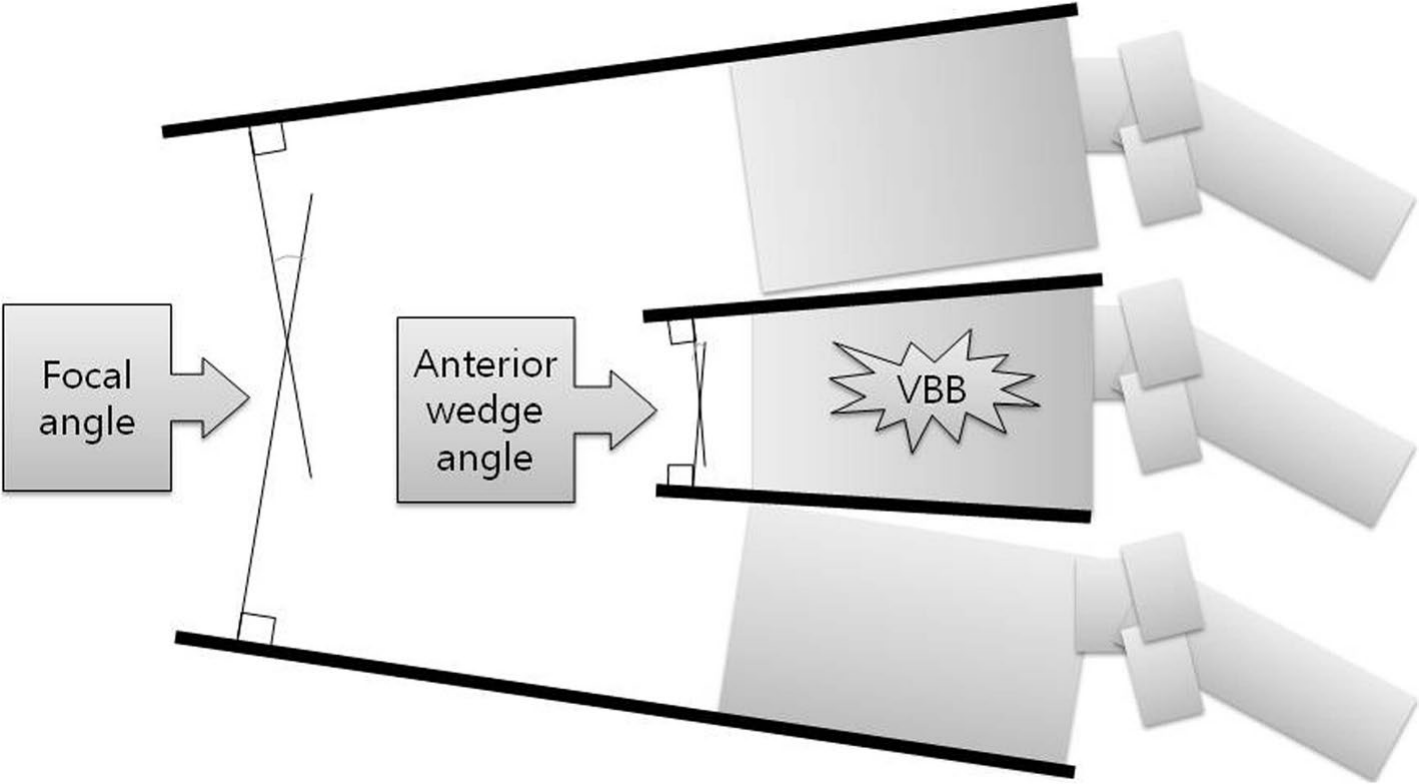
The anterior wedge angle of VBB (wedge angle) and focal angle around VBB (focal angle)

We measured the ratio of anterior to posterior cortical heights of VBB (AP ratio) and the ratio of anterior cortical heights of VBB to the average of those of cranial and caudal adjacent, non-affected vertebral bodies (ratio to adjacent spines) in the standing lateral radiographs (Fig. 3). Also, we measured the anterior wedge angle of VBB (wedge angle) and the focal angle around VBB (focal angle) in the standing lateral radiographs (Fig. 4). We compared them between the initial and final visits to check the progression of the VBB to the delayed collapse.

Whole spine standing radiographs were available in ten patients. Global sagittal alignment was analyzed by measuring the horizontal distance of the C2 plumb lines (vertical lines drawn through the center of the C2 vertebrae) and the posterior superior corner of the vertebral body of the first sacral vertebra (S1). We assigned a positive value if the C2 plumb line was anterior to the posterior superior corner of the S1 vertebral body. The C2 plumb line distance was 10.4 ± 26.5 mm (range: -38.4 to 55.6 mm). We evaluated the age of the patients, the C2 plumb line distance, the regional location of VBB of the cervical, thoracic, and lumbar spines, the etiology of VBB, and the treatment methods of the fractures as possible risk factors influencing the progression.

We evaluated the distribution of VBB within the vertebral body according to the anterior/posterior and superior/inferior areas. We evaluated patients’ pain levels with the visual analogue scale (VAS) at initial and final visits.

### Statistical analysis

Statistical analysis was performed using SPSS version 13.0. The differences in continuous variables between the two groups were examined with a paired t-test or two sample t-tests. The differences in continuous variables among the three groups of the cervical, thoracic, and lumbar regional distributions of VBB and the three groups of the etiologies of fractures were examined with ANOVA. Simple linear regression analyses were used for the effect of age and C2 plumb line distance of the patients on the difference of the ratios and the angles. Power analysis was performed by G*Power version 3.1.5 (Germany). Power was 0.95 for paired t-tests with an effect size of 0.8 and alpha error probability of 0.05. The sample size of a paired t-test should be more than 19. The statistical significance level was set at *p* < 0.05.

The intra-observer and inter-observer reliabilities were calculated using the reliability statistics by intraclass correlation (ICC) for the heights and angles. The ICC values were graded using previously described semiquantitative criteria: excellent for values in the 0.9–1.0 range, good for 0.7–0.89, fair/moderate for 0.50–0.69, low for 0.25–0.49, and poor for 0.0–0.24. The heights had 0.958 intra-observer reliability and 0.916 inter-observer reliability. The angles had 0.959 intra-observer reliability and 0.946 inter-observer reliability.

## Results

The anterior and posterior heights of a VBB were 22.6 ± 4.4 mm and 24.6 ± 5.9 mm, respectively, at the initial visit, and 22.4 ± 4.7 mm and 24.2 ± 5.8 mm, respectively, at the final visit. There was no difference in the AP ratio and the ratio to adjacent spines of the VBB between the initial and final visits (Table 2). There was no difference in the wedge angle and the focal angle of VBB between the initial and final visits, too (Table 2).

**Table 2:** The ratios and angles with VBB at the initial and final visits

	Initial	Final	*P*-value
AP ratio	0.92 ± 0.11	0.94 ± 0.10	0.267
Ratio to adjacent spines	0.97 ± 0.17	0.99 ± 0.18	0.159
Wedge angle	3.16 ± 4.76	2.14 ± 3.22	0.053
Focal angle	1.88 ± 12.99	3.06 ± 12.00	0.258

The differences between the initial and final visits in the AP ratio, the ratio to adjacent spines, the wedge angle, and the focal angle of VBB were not dependent on the age of the patients, the C2 plumb line distance, the regional location of VBB of the cervical, thoracic, and lumbar spines, the etiology of VBB, or the treatment methods of the fractures (Table 3).

**Table 3:** The differences between the initial and final visits in the ratios and the angles according to patient age, the C2 plumb line distance, the regional location of VBB of the cervical, thoracic, and lumbar spines, the etiology of VBB, and the treatment methods of the fractures (*p*-value)

	Age	C2 plumb line distance	Regional location of VBB	Etiology of VBB	Treatment methods of the fractures
AP ratio	0.624	0.252	0.685	0.501	0.544
Ratio to adjacent spines	0.690	0.339	0.315	0.674	0.281
Wedge angle	0.906	0.139	0.948	0.344	0.646
Focal angle	0.164	0.064	0.189	0.914	0.674

The anterior superior area is the most common in the distribution of VBB within the vertebral body, which is similar to the distribution of the signal change in the compression fracture (Table 4).

**Table 4:** The distribution of VBB within the vertebral body according to the anterior/posterior and superior/inferior areas

	Number	Percentage
Anterior superior	15	41.7%
Posterior superior	7	19.4%
Anterior inferior	8	22.2%
Posterior inferior	6	16.7%

The pain levels evaluated with the VAS improved from 8.9 ± 0.8 at the initial visit to 1.1 ± 0.5 at the final visit with the follow-up of 6 months.

## Discussion

The purpose of this study was to elucidate the progression of VBB in non-osteoporotic adult patients and to identify the possible risk factors influencing the progression.

Unlike compression fractures, the vertebral body with traumatic VBB did not cause the delayed collapse of the vertebral body during the follow-up period. The age of the patients, the C2 plumb line distance, the regional location of VBB, the etiology of VBB, and the treatment methods of the fractures were not risk factors contributing to the progression.

Eustace et al. found that edema in the joints is noted to be hypointense on T1-weighted and to be hyperintense on T2-weighted sequences [3]. Trauma is the most common cause of edema in the joints [3]. The edema generally resolves within 3 months and heals more rapidly in vascularized red marrow [3]. Pain with edema occurs secondary to disruption or irritation of the sensory nerves within marrow neurovascular bundles [3].

Like edema in the joint, the traumatic VBB in the spine showed a similar progression in a few studies [5–7]. The VBB on the MRI findings were retrospectively evaluated by the radiographs in the eighteen adult patients aged 38 years on average (range, 19–75) with thoracic and lumbar spine fractures, and the effect of the VBB on bone-implant abnormalities at the instrumented levels was also elucidated [5]. Anterior wedge angles were repeatedly observed [5]. They found that VBB did not cause progressive vertebral collapse or bone-implant interface failure in fracture patients [5]. Similar with the current study, they found the A3 type in twelve patients (66.7%), B1 type in one patient (5.6%), B2 type in three patients (16.7%), and B3 type in two patients (11.0%) in the study population of eighteen patients with VBB [5].

However, the number of patients in their study population was too small. In contrast to the current study, they excluded patients with cervical fractures and only included patients with thoracic or lumbar fractures. VBB were found in the thoracic (12 patients, 66.7%) and lumbar (6 patients, 33.3%) spines. Thirteen patients out of eighteen with VBB had undergone operations (72.2%), and nine patients (50.0%) had the instrumentation placed into a VBB [5]. Only nine patients were evaluated without interference of instrumentation. In addition, they did not evaluate the study population with the CT to exclude the cortical disruption of fracture. This is important because MRI has limitations compared with CT in the incomplete fractures of the pars interarticularis with marked surrounding sclerosis [8, 9]. In pediatric patients with thoracic or lumbar compression fractures, CT demonstrated high sensitivity in determining the presence or absence of fracture compared with MRI [10]. MRI had a sensitivity of 100%, specificity of 97%, negative value of 75%, and positive predictive value of 100% in detecting spine injury using CT as the standard for osseous injury [11]. Also, they did not perform bone densitometry in order to exclude patients with osteoporotic fractures. Their study population might include patients with osteoporosis. Finally, they did not evaluate the effect of age, the sagittal balance of the C2 plumb line distance, the regional location of VBB, the etiology of VBB, or the treatment methods of the fractures, which could be possible risk factors making the progression.

Yokoyama et al. found six children, between the ages of 9 and 13, who had a single VBB by using MRI. At the on-month follow-up, the signal changes seen on MRI had disappeared [6]. Scheunemann et al. reported a case series of 20 children with VBB found on MRI in the German literature [7]. At the final follow-up MRI, there was no collapse nor bruise of the vertebral body [7]. However, both studies did not evaluate adult patients.

Thirty-nine patients (5.8%) with VBB were identified in the current study. Green et. al found 57% of VBB in 127 traumatic patients [4]. Unfortunately, they did not evaluate the VBB with CT to rule out the definite fracture. The low incidence of VBB in the current study might rule out the fracture with CT.

As with any study, our investigation had limitations. The sample population was small. We have a plan to recruit a larger study population in the future. Second, we did not provide information about the resolution of VBB based on the MRI at the final visit and the time for the VBB to be resolved due to the cost problem of MRI. Third, the study population had spinal fractures in other spinal levels concurrent with VBB. The pain evaluated with the VAS in the current study was caused by both spinal fracture and VBB. Fourth, the current study is a case-series study. It would be better to perform the study with the design that includes a case–control with the spinal fracture patients matched with and without VBB or the design of a multifactorial analysis with more parameters. However, the number of patients with VBB was limited in the current study. We have a plan to recruit a larger sample size to make a case–control study or a multifactorial analysis in the future. Despite these limitations, to the best of our knowledge, this study represents the report with a larger study population and stricter inclusion criteria based on CT, MRI, and bone densitometry to elucidate the fate of a traumatic VBB in non-osteoporotic adult patients with spinal fratures.

## Conclusions

The vertebral body with traumatic VBB found in the adult patients with non-osteoporotic spinal fractures of AO classification A or B types did not develop collapse. In clinical practice, the compression fracture might be misdiagnosed as the VBB in the initial evaluation if the VBB leads to collapse during the follow-up. If the collapse occurs, it needs to be diagnosed as a spinal fracture rather than a VBB. The current study might help address economic and legal issues because the orthoses necessary for patients with spinal fractures are not necessary for patients with VBB. In addition, the disabilities diagnosed for or the compensations provided to patients with spinal fractures are not available to patients with VBB.

## Data Availability

All of the data supporting findings are contained within the manuscript. Disaggregated data cannot be made publicly available because of ethical restrictions. Please contact the corresponding author.
